# Preventive effect of salmon sperm DNA on acute carbon tetrachloride‐induced liver injury in mice through Nrf2/ARE and mitochondrial apoptosis pathway

**DOI:** 10.1002/fsn3.3109

**Published:** 2022-10-26

**Authors:** Xinyi Huang, Xu Chu, Yingying Tian, Yuhan Xue, Lei Zhang, Jing Li, Hu Hou, Ping Dong, Jingfeng Wang

**Affiliations:** ^1^ College of Food Science and Engineering Ocean University of China Qingdao China

**Keywords:** carbon tetrachloride liver injury, inflammation, mitochondrial apoptosis pathway, Nrf2/ARE oxidation pathway, salmon sperm DNA

## Abstract

Liver injury refers to the damage of liver function, which will seriously harm the body’s health if it is not prevented and treated in time. Sporadic researches have reported that ingestion of DNA has a hepatoprotective effect, but its effect and mechanism were not clarified. The purpose of this study was to explore the preventive effect and mechanism of salmon sperm DNA on acute liver injury in mice induced by carbon tetrachloride (CCl_4_). Six‐week‐old ICR (Institute of Cancer Research) male mice were used to establish a liver injury model by injecting with 4% CCl_4_, silymarin, and three different concentrations of DNA solutions were given to mice by gavage for 14 days. The histological and pathological changes in the liver were observed. The levels of alanine aminotransferase (ALT) and aspartate aminotransferase (AST) in serum and the levels of oxidative and antioxidant markers such as malondialdehyde (MDA), superoxide dismutase (SOD), glutathione peroxidase (GSH‐Px), and glutathione (GSH) in liver tissue were determined. The levels of interleukin‐6 (IL‐6) and tumor necrosis factor‐α (TNF‐α) were detected by enzyme‐linked immunosorbent assay (ELISA), and hepatic oxidative stress and apoptosis‐related markers were determined by western blotting. The results showed that compared with the model group, the DNA test group significantly improved the liver pathological changes and the level of liver function, regulated liver oxidative stress, reduced hepatocyte apoptosis, and decreased the levels of inflammatory factors such as TNF‐α and IL‐6. Compared with the silymarin group, the high dose of DNA was even more effective in preventing liver injury. In conclusion, salmon sperm DNA has a potential protective effect against acute liver injury induced by CCl_4_, which is achieved by regulating the Nrf2/ARE (nuclear factor erythroid 2 (NF‐E2)‐related factor 2/antioxidant responsive element) oxidative stress pathway and mitochondrial apoptosis pathway.

## Graphical abstract

The purpose of this study was to explore the preventive effect and mechanism of salmon sperm DNA on acute liver injury in mice induced by carbon tetrachloride. The study will provide a reference basis for nutrition evaluation, function mining and accurate utilization of aquatic DNA.

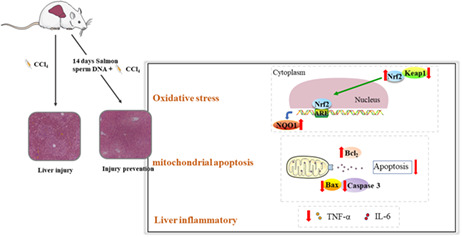

## 1 INTRODUCTION

Liver is considered to be an important organ for the metabolism of drugs and chemicals (Rowland et al., 2013), and its important role is to detoxify (Vishwakarma et al., 2019), dysfunction of detoxification, and the metabolic pathways of the liver injury (Ruart et al., 2019). Liver injury refers to the damage of liver function caused by a series of blows to the liver, such as toxic damage, alcohol abuse, viral infection, and metabolic disorders (Di Paola et al., 2022), if it is not prevented and treated in time, it may progress to more serious hepatitis (Irshad et al., 2019), liver cirrhosis, and even liver cancer (Cordero‐Espinoza & Huch, 2018), which is seriously harmful to body health. So far, few drugs have been approved to alleviate human liver disease (Freedman et al., 2011), and most of them have hidden dangers of side effects (Liu et al., 2014; Ma et al., 2020). Therefore, screening the functional ingredients of natural food sources that can regulate liver function and improve liver injury from the perspective of diet has been paid more and more attention, and it has become one of the most concerned research hotspots in the field of nutrition and health in recent years.

As an important molecule for biological composition (Sánchez‐Pozo & Gil, 2002), nucleic acids have been reported to have a variety of beneficial physiological functions, such as antioxidation (Wang et al., 2008), improving immunity (Cheng et al., 2018; Xu et al., 2013), liver function (Kojima‐Yuasa et al., 2016; Sauer et al., 2012), and growth performance and nutrient utilization (Waititu et al., 2015), repairing intestinal injury (Meng et al., 2017) and regulating intestinal flora (Sauer et al., 2012). However, the use of natural macromolecular nucleic acids in medicine and food processing is limited because of their insolubility in water and low bioavailability. There are few studies on their physiological functions, and at present, they are mainly focused on single nucleotides. However, the diet contains more macromolecular nucleic acids from cellular components (Pickering et al., 1998). It has been found that a nucleoside–nucleotide mixture can improve liver injury in rats (Shohei et al., 1988) and is an effective nutritional supplement for metabolism in cirrhotic rats after partial hepatectomy (Usami et al., 1996). Although there are some reports on the improvement of the liver by a direct intake of DNA, the researches were still at the level of the phenomenon, and the mechanisms’ clarification was lacking. It affected the mining and application of nucleic acids with a hepatoprotective effect.

Liver injury will trigger necrosis or apoptosis, amplifying the pro‐inflammatory response (Fusco et al., 2020), and it is closely related to oxidative stress and mitochondrial dysfunction (Zhang et al., 2021). It has been reported that the increased oxidative stress characteristic of liver dysfunction leads to damage to mitochondrial arrogance and accumulation of dysfunction (Di Paola et al., 2022). Nrf2 (nuclear factor erythroid 2 (NF‐E2)‐related factor 2) is a major regulator of protective antioxidant responses. Under physiological conditions, it is sequestered in the cytoplasm by its inhibitor Kelch‐like ECH‐associated protein 1 (Keap1), which mediates the proteasomal degradation of Nrf2 (Robledinos‐Antón et al., 2019). Once cellular oxidative stress occurs, Keap1 undergoes conformational modification to prevent Nrf2 degradation and allow its accumulation in the nucleus (Cordaro et al., 2020). Bcl‐2‐associated X protein (Bax) is a typical pro‐apoptotic protein in cytosols, which can be transported to mitochondria to induce apoptosis, while B‐cell lymphoma 2 (Bcl‐2) is an anti‐apoptotic protein to inhibit Bax‐induced apoptosis, Caspase 3 is a key protease in the process of apoptosis and a key effector downstream of many apoptotic pathways, and these indexes can reflect the degree of apoptosis (Jia et al., 2021).

Carbon tetrachloride (CCl_4_) is a well‐known hepatotoxin that can induce liver injury through a variety of mechanisms, including oxidative stress, inflammation (Huang et al., 2012; Ning et al., 2018; Reyes‐Gordillo et al., 2017), and apoptosis (Jia et al., 2021). In this study, a mouse model of acute liver injury induced by CCl_4_ was established (Masuda, 2006). Naturally existing salmon sperm DNA was given orally to mice with amino acid defined feed (lack of nucleic acids) in our study. The preventive and protective effects of salmon sperm DNA on liver injury were preliminarily identified and compared, and its protective mechanism was explored from many aspects such as oxidation, inflammation, and apoptosis.

## 2 MATERIALS AND METHODS

### 2.1 Experimental animal

In this study, we used a classical model of mice treated with CCl_4_ as a model of CCl_4_‐induced acute hepatic damage. Male ICR (Institute of Cancer Research) mice (age 6 weeks and weight 20–22 g) were obtained from Jinan Pengyue Co. Ltd.

### 2.2 Drugs and reagents

Sodium deoxyribonucleic acid salt (derived from salmon sperm) was purchased from Sigma. The mass concentration of DNA in the sodium deoxyribonucleic acid salt of 0.02 mg/mL was determined by NanoDrop to be 1.25 mg DNA/mg sodium deoxyribonucleic acid sodium salt. Sodium carboxymethyl cellulose (0.5%) was used to prepare suspensions containing DNA with final concentrations of 10 mg/kg (low dose), 50 mg/kg (medium dose), and 300 mg/kg (high dose), respectively. Olive oil was purchased from Hangzhou Guanlai Trading Co. Ltd.; CCl_4_ was purchased from Sinopharm Chemical Reagent Co. Ltd, diluted with olive oil. Aspartate aminotransferase (AST) kit, alanine aminotransferase (ALT) kit, superoxide dismutase (SOD) kit, malondialdehyde (MDA) kit, glutathione peroxidase (GSH‐Px) kit, and glutathione (GSH) kit were all purchased from Nanjing Jiancheng Biological Engineering Co. Ltd, while ELISA kits TNF‐α and IL‐6 were purchased from Thermo Fisher Scientific, USA. TUNEL (terminal deoxynucleotidyl transferase (TdT)‐mediated dUTP nick end labeling) apoptosis kit and bicinchoninic acid (BCA) protein concentration determination kit were purchased from Beijing Solarbio Science & Technology Co. Ltd. β‐Actin antibody was purchased from Affinity and other antibodies were purchased from ProteinTech Group.

### 2.3 Methods

#### 2.3.1 Establishment of the mouse model of acute liver injury

After 5 days of adaptive feeding, the mice were rectum divided into 6 groups, with 6 mice in each group: Normal control group (Normal), model control group (Model), positive control group (Positive), DNA low dose group (L‐DNA), DNA medium dose group (M‐DNA), and DNA high dose group (H‐DNA), 6 mice in each group. The mice in each group were marked with the corresponding number, during the experiment, the status of mice in each group was observed every day, and the mice were weighed and recorded every 3 days. The experimental period was 14 days (intragastric dose of 0.01 ml/g). All mice in the normal control group and model control group were given 0.5% sodium carboxymethyl cellulose solution, and the positive control group was given silymarin solution (100 mg/kg). The rest of the mice were given daily intragastric administration of DNA solution according to a low dose of 10 mg/kg, a medium dose of 50 mg/kg, and a high dose of 300 mg/kg. Eight hours after the last intragastric administration, except for the normal group, all the mice were intraperitoneally injected with 4% CCl_4_ olive oil solution (the intraperitoneal injection volume was 10 μl/g). The mice were killed after fasting for 16 h: take out the eyeballs of the mice and collect the blood, centrifuge the serum, and store it at −20°C. Take the liver tissue quickly, wash it with cold saline, take pictures, then take about 0.1 g in the middle of the largest lobe of the liver and fix it in 4% paraformaldehyde solution, and then put the rest of the liver tissue into liquid nitrogen first, and finally store all the tissues in the refrigerator at −80°C.

#### 2.3.2 Hepatic histopathology

The mouse liver tissue was first embedded in paraffin wax, samples were sectioned and stained with hematoxylin–eosin (H&E), Taking pictures under an optical microscope and observe.

#### 2.3.3 TUNEL assay

Cell apoptosis of the liver tissues was detected using an in situ cell death detection kit following the manufacturer's instructions. Paraffin sections were dewaxed, repaired, broken, reagents 1 and 2 added, DAPI (4′,6‐diamidino‐2‐phenylindole) restained nuclei, sealed, observed under a fluorescence microscope, and photographed.

#### 2.3.4 Determination of biochemical indexes

Centrifuged fractionated serum was directly used to determine the levels of ALT and AST according to the kit. About 0.1 g of liver tissue was added to 9 times the volume of saline in the ratio of 1:9 to prepare a 10% homogenate, and the supernatant was taken after centrifugation, and the protein concentration (mg/mL) was measured by the BCA kit. Then, the levels of SOD, MDA, GSH‐Px, and GSH were measured according to the kit instructions.

#### 2.3.5 Western blotting

Total protein was extracted from liver tissue with lysis buffer (mixed with phenylmethylsulfonyl fluoride (PMSF) and radioimmunoprecipitation assay (RIPA) at 1: 100), protein quantification was used a BCA protein concentration determination kit, the total protein was separated by sodium dodecyl sulfate‐polyacrylamide gel electrophoresis (SDS‐PAGE) and transferred to a nitrocellulose (NC) membrane. And then, put the NC membrane into Tris‐buffered saline with Tween 20 (TBST) containing 5% skim milk and wash it with TBST for 3 times (10 min at a time), and incubated in a special primary antibody overnight. The membrane was washed three times the next day and then incubated with the secondary antibody for 2 h at room temperature.

#### 2.3.6 Enzyme‐linked immunosorbent assay (ELISA)

The levels of tumor necrosis factor‐α (TNF‐α) and interleukin‐6 (IL‐6) in liver tissue were analyzed by the ELISA kits TNF‐α(BMS607‐3), IL‐6(BMS603‐2) on Thermo Fisher Scientific, USA, according to the manufacturer’s instructions, respectively.

#### 2.3.7 Statistical processing

IBM SPSS Statistic 22.0 statistical software was used for data analysis. One‐way analysis of variance (ANOVA) was used for the comparison of means between groups, and data results were expressed as mean ± standard error (mean ± SEM). * indicates a significant difference for the model control group compared to the normal control group (*p* < .05), ** indicates a highly significant difference (*p* < .01), ^#^ indicates a significant difference for the subject group compared to the model control group (*p* < .05), and ^##^ indicates a highly significant difference (*p* < .01).

## 3 RESULTS

### 3.1 Effects of salmon sperm DNA on the appearance and morphology of mice with acute liver injury

**FIGURE 1:**
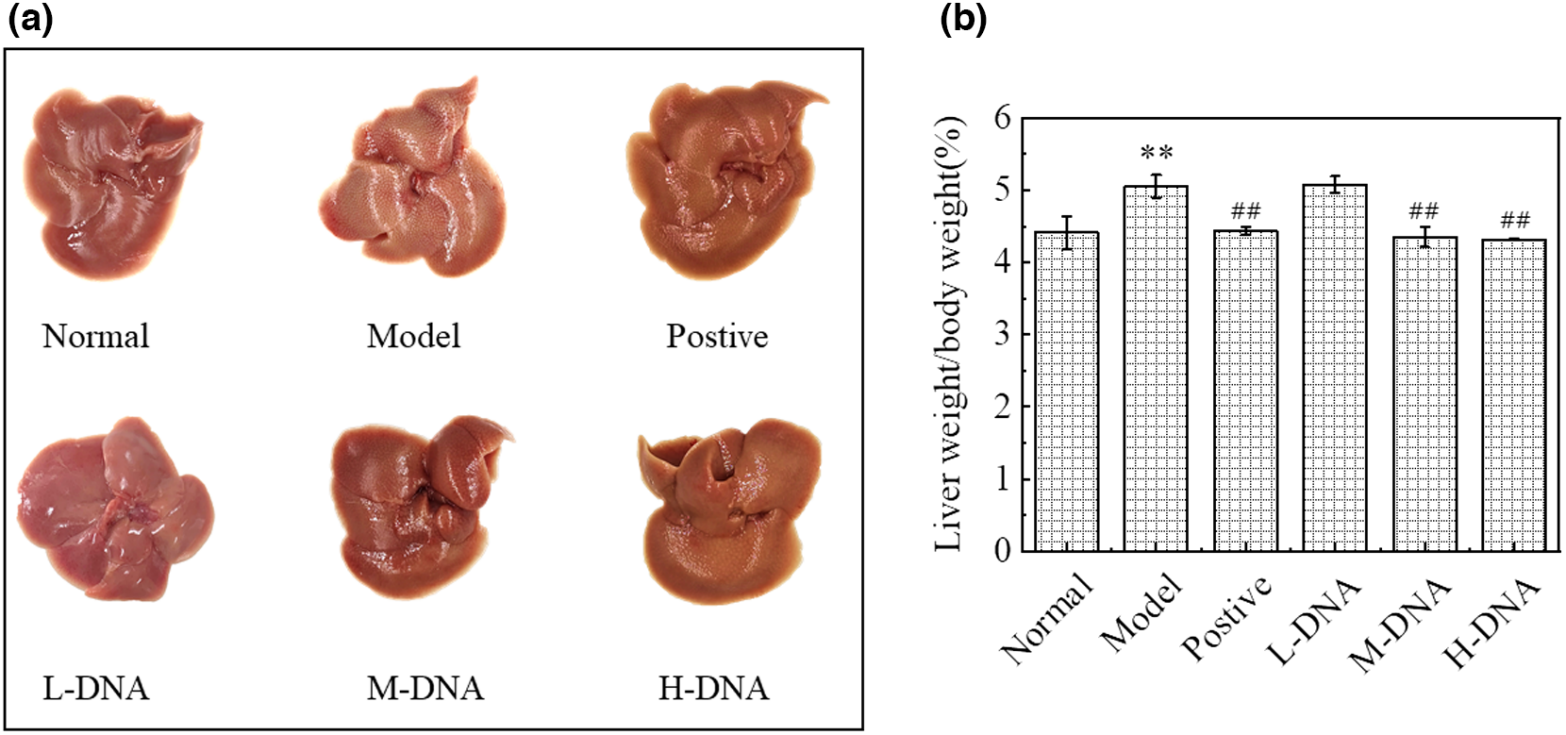
Effects of DNA on liver morphology and organ index after carbon tetrachloride (CCl_4_)‐induced acute liver injury in mice. (a) Effect of DNA on liver morphology in mice with acute liver injury. (b) Effect of DNA on liver index (%) in mice with acute liver injury. * *p* < .05 vs Normal group, ** *p* < .01 vs Normal group; # *p* < .05 vs Model group, ## *p* < .01 vs Model group (*n* = 6)

To observe the newly dissected mouse liver with the naked eye, as shown in Figure 1a: the liver of normal mice showed reddish brown color, smooth appearance, and neat edges. In the Model group injected intraperitoneally with CCl_4_, the liver was white in color, with small white particles, fragile texture and adhesion, the surface was not smooth and the edge was congested, and the volume was obviously swollen. The liver of the model group injected intraperitoneally with CCl_4_ was light reddish brown, with small white particles, fragile texture and adhesion, the surface was not smooth and the edge was congested, and the volume was obviously enlarged. The lighter color and injury of liver could be repaired in the positive group, the DNA medium dose (M‐DNA) group, and the DNA high dose (H‐DNA) group, which was closer to the normal group. During the feeding period, the body weight and food intake of mice in each group were normal. The changes of body weight and organ index of mice after a large amount of intraperitoneal injection of CCl_4_ are shown in Figure 1b: compared with the Normal group, the liver index of the Model group increased by about 14.54% (*p* < .01), while compared with the Model group, after the intervention of M‐DNA group and H‐DNA group (50 mg/kg·bw and 300 mg/kg·bw), the liver index decreased by 13.85% and 14.62%, respectively (*p* < .01). The results showed that DNA could inhibit the increase of liver index induced by intraperitoneal injection of CCl_4_.

### 3.2 Effects of salmon sperm DNA on serum ALT and AST levels in mice with acute liver injury induced by CCl_4_

**FIGURE 2:**
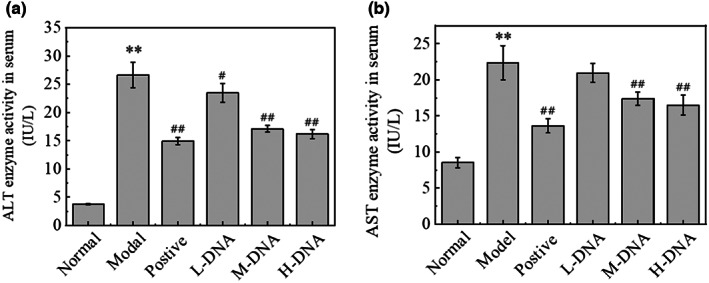
Serum alanine aminotransferase (ALT) and aspartate aminotransferase (AST) levels in mice with carbon tetrachloride (CCl_4_) liver injury. (a) The inhibitory effects of DNA on serum ALT; (b) the inhibitory effects of DNA on serum AST. * *p* < .05 vs Normal group, ** *p* < .01 vs Normal group; # *p* < .05 vs Model group, ## *p* < .01 vs Model group (*n* = 6)

As shown in Figure 2, compared with the Normal group, the activities of ALT and AST in the serum of the Model group were significantly higher than those of the Normal group (*p* < .01). Compared with the Model group, salmon sperm DNA group decreased the activities of serum ALT and AST in a dose‐dependent manner, and both of them showed the most significant results of the H‐DNA group, the proportion of reduction being as high as 39.31% (*p* < .01) and (26.24%), respectively.

### 3.3 Effect of salmon sperm DNA on liver histopathology in mice with acute liver injury induced by CCl_4_

**FIGURE 3:**
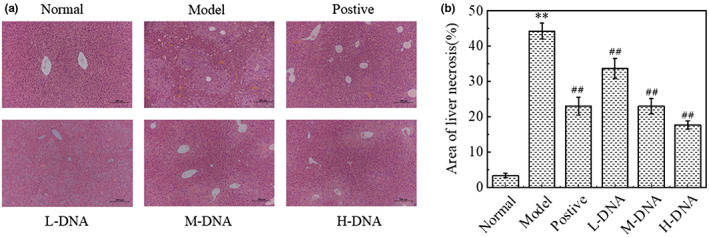
Microscopic observation of hematoxylin and eosin (HE) section and quantification of necrotic area in mice with acute carbon tetrachloride (CCl_4_) liver injury (10×). (a) Micrographs of HE sections (200 μM); (b) quantitative maps of the area of the cell necrosis region. * *p* < .05 vs Normal group, ** *p* < .01 vs Normal group; # *p* < .05 vs Model group, ## *p* < .01 vs Model group (*n* = 6)

In order to further evaluate the protective effect of salmon sperm DNA on acute liver injury induced by CCl_4_, liver histopathology was examined. As shown in Figure 3, the hepatocytes in the Normal group arranged radially along the central vein, and the lobular structure was complete and clear, while in the Model group, the hepatocytes arranged in a disorderly manner, the structure of hepatic lobules was blurred, a large number of necrotic foci could be seen, accompanied with symptoms of inflammatory infiltration, and the characteristics of liver injury were obvious. Compared with the Model group, the silymarin positive hepatocytes arranged slightly neatly, the necrotic area decreased, and only a small amount of inflammatory cell infiltration was seen. The M‐DNA group and L‐DNA group could restore the structure of liver tissue and significantly reduce the area of necrotic area in a dose‐dependent manner (*p* < .01), and the symptoms of inflammatory infiltration and liver injury were significantly reduced.

### 3.4 Effect of salmon sperm DNA on hepatocyte apoptosis in mice with liver injury induced by CCl_4_

**FIGURE 4:**
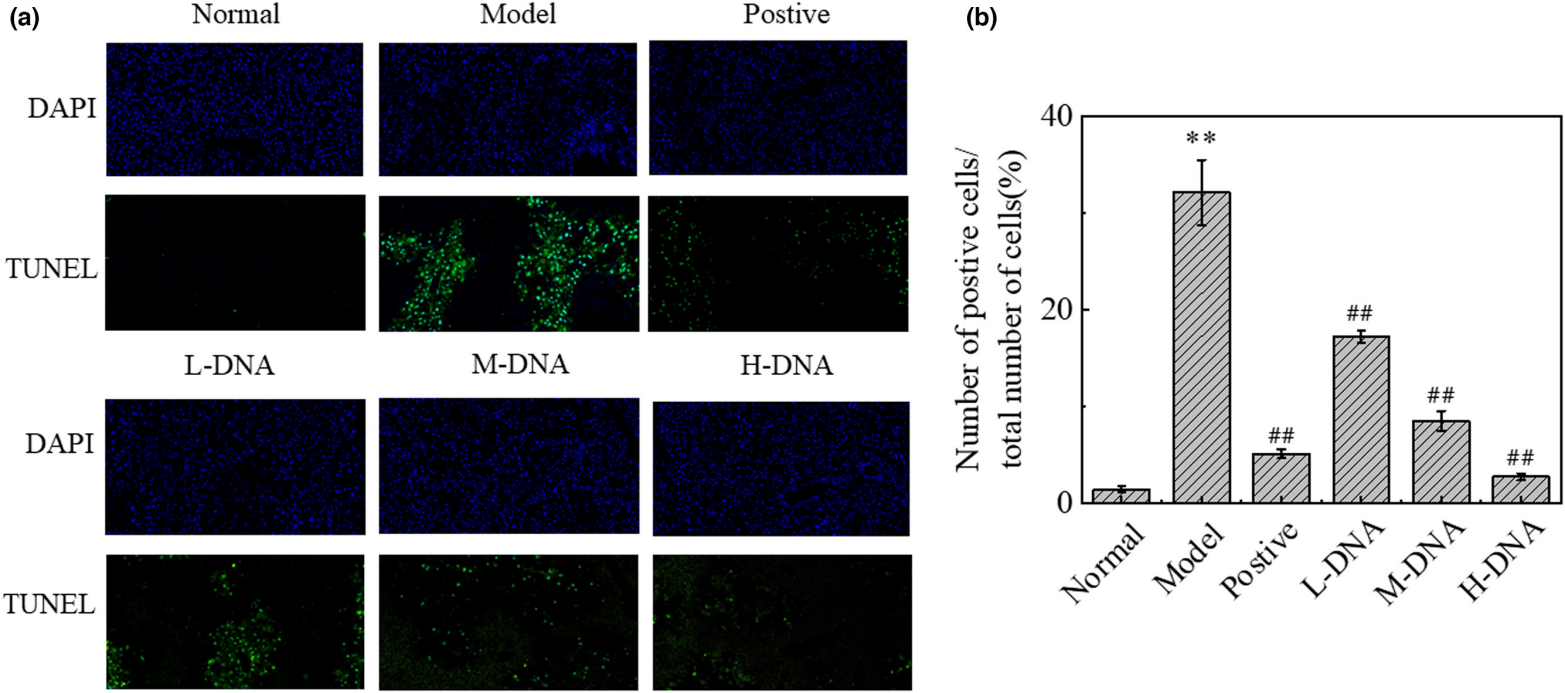
Effect of DNA on carbon tetrachloride (CCl_4_)‐induced cell apoptosis in liver tissues. (a) Representative TUNEL (terminal deoxynucleotidyl transferase dUTP nick end labeling)‐stained sections showing apoptosis in the liver tissue of mice from Normal, Model, Positive, DNA low dose (L‐DNA), DNA medium dose (M‐DNA), and DNA high dose (H‐DNA) treated groups. And the foci (green) were quantified by an ImageJ software. (b) The number of positive total number of cells (%). * *p* < .05 vs Normal group, ** *p* < .01 vs Normal group; # *p* < .05 vs Model group, ## *p* < .01 vs Model group (*n* = 6). Original magnification, ×20

In order to clarify the effect of CCl_4_ on acute liver injury and its inhibitory effect on hepatocyte apoptosis, we performed TUNEL (terminal deoxynucleotidyl transferase dUTP nick end labeling) staining to evaluate the protective effect of salmon sperm DNA on hepatocyte apoptosis induced by CCl_4_. TUNEL staining showed that the percentage of apoptotic cells in CCl_4_‐induced mice was higher than that in normal mice, salmon sperm DNA group decreased the number of apoptosis in a dose‐dependent manner (*p* < .01) Figure 4.

### 3.5 Effect of salmon sperm DNA on the expression of Bax, Bcl‐2, and Caspase 3 in liver tissues of mice with CCl_4_ ‐induced liver injury

**FIGURE 5:**
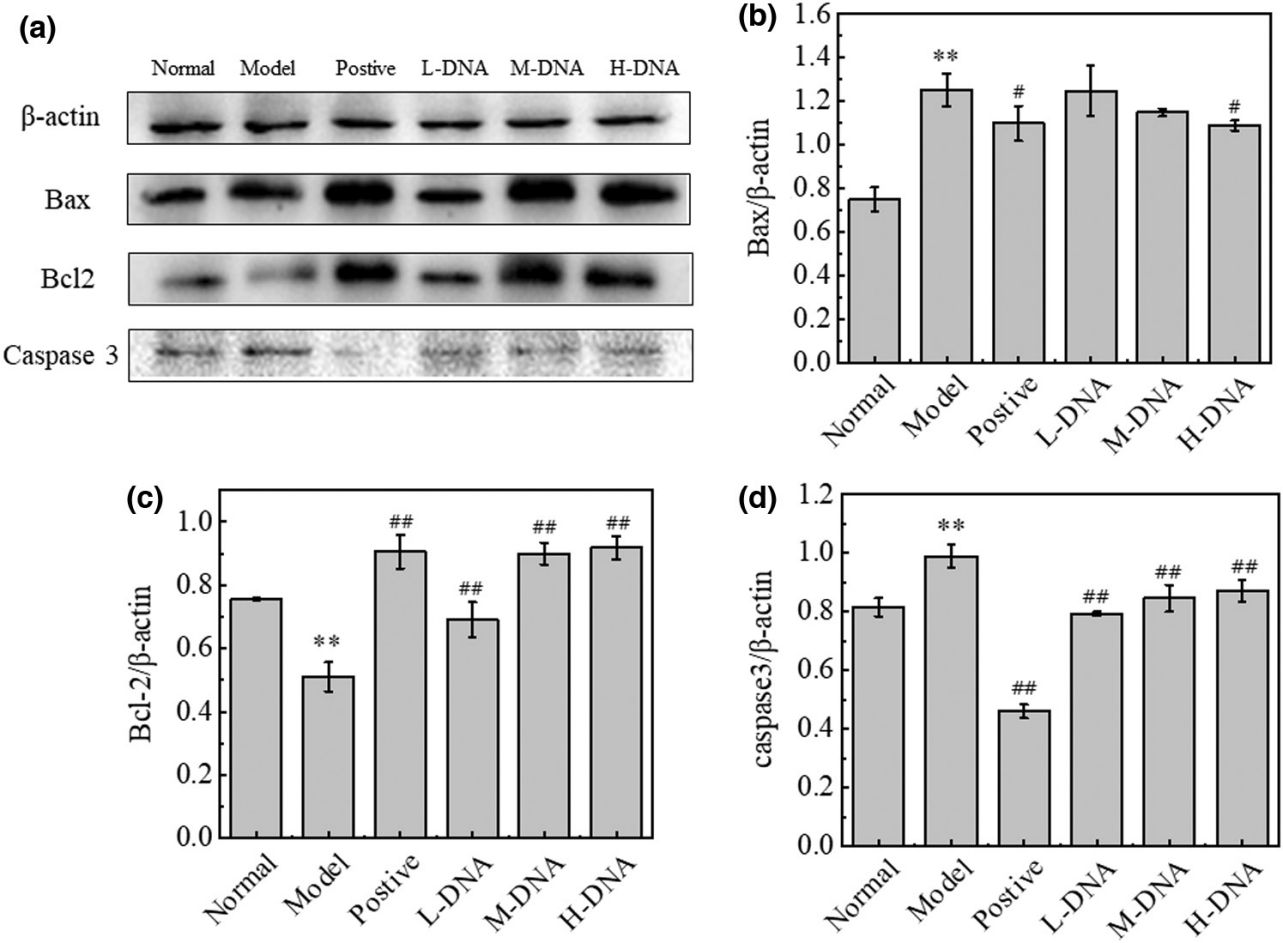
Effect of salmon sperm DNA on the expression of Bcl‐2‐associated X protein (Bax), B‐cell lymphoma 2 (Bcl‐2), and Caspase 3 in liver tissues of mice with carbon tetrachloride (CCl_4_)‐induced liver injury. Bax, Bcl‐2, and Caspase 3 protein expression was examined in the liver tissue of mice from Normal, Model, Positive, DNA low dose (L‐DNA), DNA medium dose (M‐DNA), and DNA high dose (H‐DNA) treated groups. (a) Immunoblot images from representative experiments. Changes in the liver tissue expression of (b) Bax, (c) Bcl‐2, and (d) Caspase 3. Values represent the mean ± SD (*n* = 6). * *p* < .05 vs Normal group, ** *p* < .01 vs Normal group; # *p* < .05 vs Model group, ## *p* < .01 vs Model group

Western blotting was used to detect the expression of apoptosis‐related genes Bcl‐2, Bax, and Caspase 3 in liver tissue to explore the mechanism of anti‐apoptosis effect of salmon sperm DNA. Compared with the Model group, salmon sperm DNA group significantly upregulated the expression of Bcl‐2 (*p* < .01) and downregulated the expression of Caspase 3 (*p* < .01) and Bax Figure 5.

### 3.6 Effect of Salmon sperm DNA on the expression of SOD, MDA, GSH‐PX, and GSH in liver tissues of mice with CCl_4_ ‐induced liver injury

**FIGURE 6:**
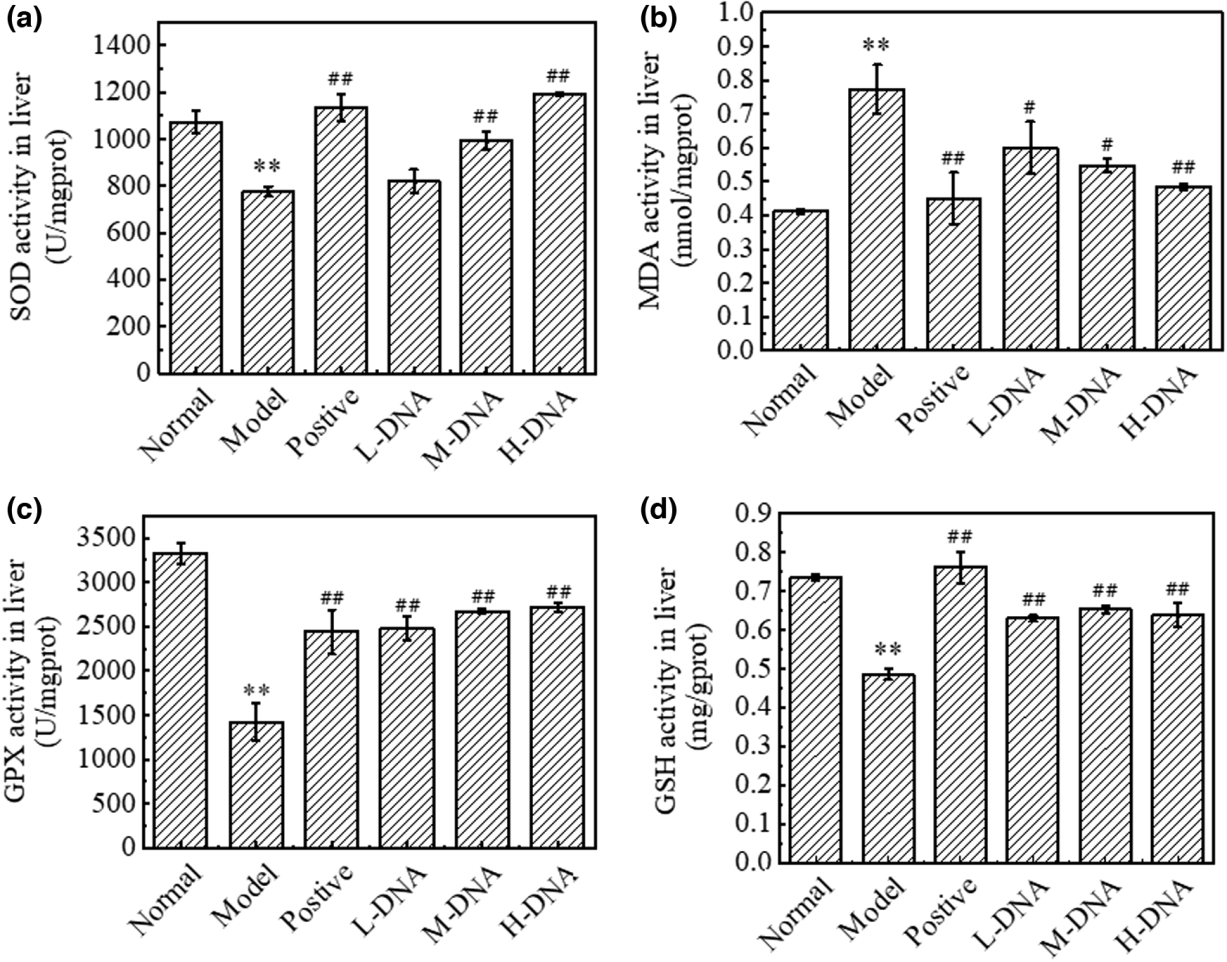
Effect of salmon sperm DNA on the expression of superoxide dismutase (SOD), malondialdehyde (MDA), glutathione peroxidase (GSH‐Px), and glutathione (GSH) in liver tissues of mice with carbon tetrachloride (CCl_4_)‐induced liver injury. (a) Hepatic SOD activity. (b) Hepatic MDA content. (c) Hepatic GPx activity. (d) Hepatic GSH content. Values represent the mean ± SD (*n* = 6). * *p* < .05 vs Normal group, ** *p* < .01 vs Normal group; # *p* < .05 vs Model group, ## *p* < .01 vs Model group

Lipid peroxidation is an important pathological process of liver injury. CCl_4_ poisoning leads to the increase of reactive oxygen species (ROS) in hepatocytes, which leads to the increase of GSH consumption and lipid peroxidation. The final product of lipid peroxidation is MDA, which is a marker of oxidative stress. At the same time, oxidative stress can also lead to a decrease in the activities of SOD and GSH‐Px. As shown in Figure 6, compared with the Normal group, the activities of SOD, GSH, and GSH‐Px in the liver of the Model group decreased significantly (*p* < .01), while the content of MDA increased significantly (*p* < .01), indicating that CCl_4_ caused serious lipid peroxidation in the liver. Compared with the Model group, the Positive group could significantly increase the activities of SOD, GSH, and GSH‐Px (*p* < .01), and reduce the content of MDA significantly (*p* < .01). The middle and high doses of DNA had a tendency to significantly increase the level of SOD, and all the groups of DNA could significantly increase the activities of GSH‐Px and GSH (*p* < .01). At the same time, compared with the Model group, high dose of DNA could reduce the content of MDA in liver (*p* < .01). The results showed that the high dose group of DNA had better ability to prevent liver lipid peroxidation in a dose‐dependent manner.

### 3.7 Effect of salmon sperm DNA on the expression of Nrf2, Keap1, and NQO1 in liver tissues of mice with CCl_4_ ‐induced liver injury

Oxidative stress serves as an important link in liver injury caused by CCl_4_, and in recent years, it has been found that Nrf2/ARE pathway is an important pathway of endogenous antioxidant stress. After the activation of the Nrf2/ARE pathway, the expression of a variety of downstream protective genes is activated, including antioxidant protein genes, detoxification enzyme genes, and anti‐inflammatory factor genes (Itoh et al., 1999). In a normal state, Nrf2 is bound to its repressor protein keap1 (Kelch‐like ECH‐associated protein 1) in the cytoplasm (Taguchi et al., 2011) and is not transcriptionally active, when conformational modification of keap1 by electrophilic substances such as free radicals leads to the dissociation of Nrf2 from it (Canning et al., 2015) and an increase in the number of Nrf2 it incorporates into the nucleus and binds to the ARE (antioxidant responsive element) promoter sequence (Copple et al., 2008), causing antioxidant proteins such as SOD (superoxide dismutase) and NQO1 (NADH dehydrogenase, Quinone 1, reduction‐dependent coenzyme) (Hafez et al., 2014) to be efficiently expressed, and these enzymes are involved in the protection of heart, brain, lung, liver, and kidney tissue cells against oxidative stress damage.

**FIGURE 7:**
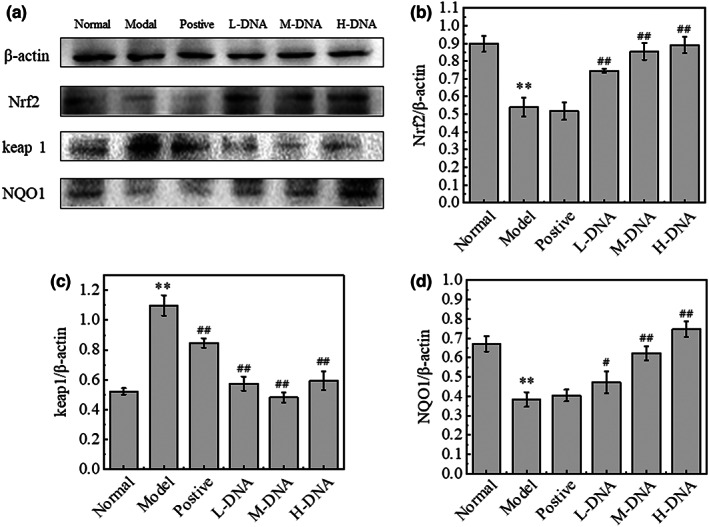
Effect of salmon sperm DNA on the expression of nuclear factor erythroid 2 (NF‐E2)‐related factor 2 (Nrf2), Kelch‐like ECH‐associated protein 1 (Keap1), and NADH dehydrogenase, Quinone 1 (NQO1) in liver tissues of mice with carbon tetrachloride (CCl_4_)‐induced liver injury. Nrf2, Keap1, and NQO1 protein expression was examined in the liver tissue of mice from Normal, Model, Positive, DNA low dose (L‐DNA), DNA medium dose (M‐DNA), and DNA high dose (H‐DNA) treated groups. (a) Immunoblot images from representative experiments. Changes in the liver tissue expression of (b) Nrf2, (c) Keap1, and (d) NQO1. * *p* < .05 vs Normal group, ** *p* < .01 vs Normal group; # *p* < .05 vs Model group, ## *p* < .01 vs Model group (*n* = 6)

The expression of Nrf2/ARE pathway protein detected by western blot is shown in Figure 7: compared with the Normal group, the expression of keap1 protein in the Model group increased significantly (*p* < .01), while the protein expression of Nrf2 and NQO1 decreased significantly (*p* < .01). In contrast, compared with the Model group, the expression of keap1 protein decreased significantly (*p* < .01), while the expression of Nrf2 and NQO1 protein increased significantly after the intervention of low, middle, and high doses of DNA in advance.

### 3.8 Regulation of salmon sperm DNA on the levels of liver inflammatory cytokines TNF‐α and IL‐6 in mice with acute liver injury

**FIGURE 8:**
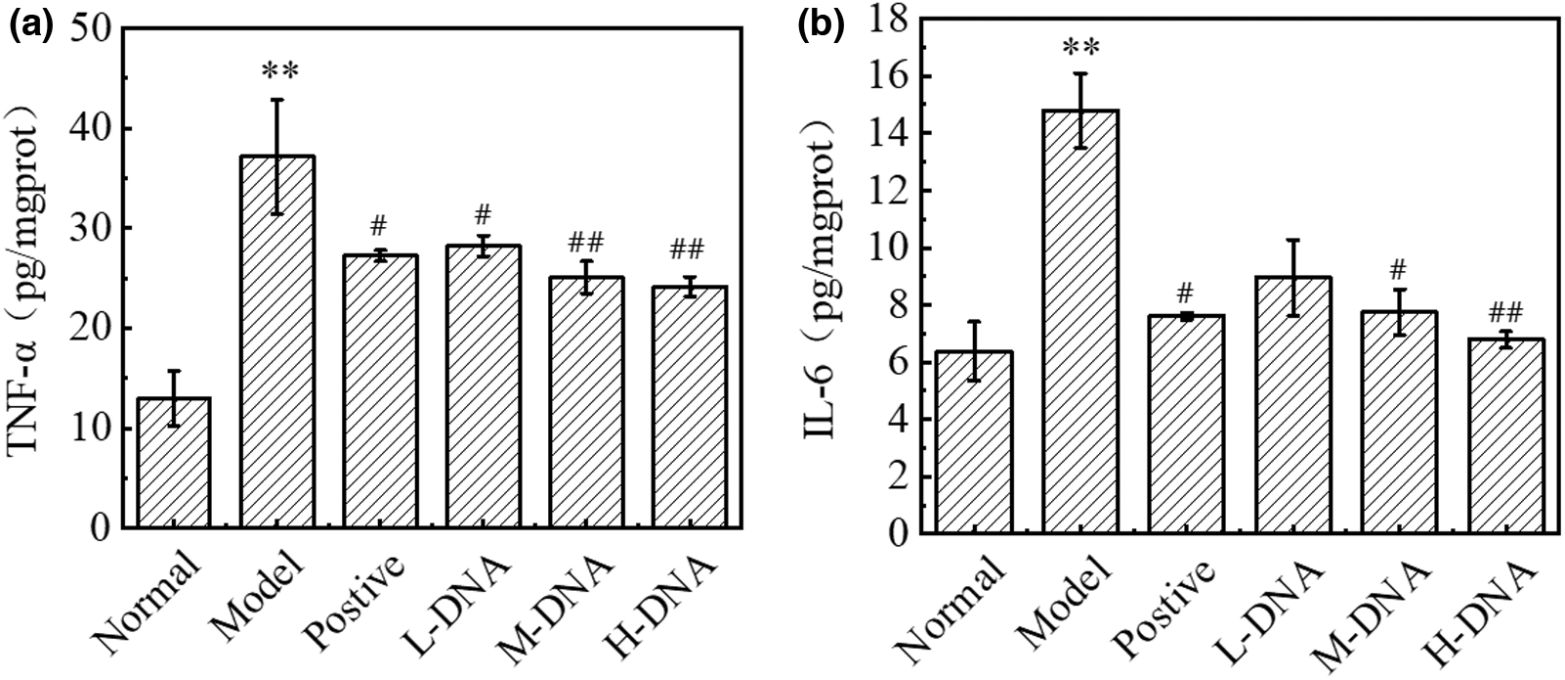
(a) Hepatic tumor necrosis factor‐α (TNF‐α) and (b) hepatic interleukin‐6 (IL‐6) level were measured by enzyme‐linked immunosorbent assay (ELISA). * *p* < .05 vs Normal group, ** *p* < .01 vs Normal group; # *p* < .05 vs Model group, ## *p* < .01 vs Model group (*n* = 6)

As shown in Figure 8, compared with the Normal group, the levels of IL‐6 and TNF‐α in the liver of mice in the CCl_4_ model control group increased significantly (*p* < .01). Compared with the Model group, salmon sperm DNA could significantly reduce the levels of IL‐6 and TNF‐α in the liver of mice in a dose‐dependent manner, and the effect was even better than the Positive group. It is suggested that salmon sperm DNA has a good anti‐inflammatory effect.

## 4 DISCUSSION

It has been demonstrated that CCl_4_ has a direct membrane solubilizing effect on the cell membrane after entering the organism, leading to Ca^2+^ inward flow and homeostatic dysregulation, causing hepatomegaly and increased liver coefficients (Liu et al., 2018), including similar effects on other tissues such as spleen and kidney (Zhang et al., 2020). When the hepatocyte membrane is disrupted, a variety of intracellular enzymes can cross the cell membrane to the hepatic sinusoidal gap and enter the blood. ALT and AST, as intracellular enzymes, are only released into the blood in very small amounts under normal conditions, and if the serum levels of ALT and AST are significantly elevated (Sato et al., 2014), it suggests necrosis of hepatocytes and liver damage. In this study, the middle and high doses of salmon sperm DNA could reduce the liver index and improve the swelling significantly. The observation of liver pathological hematoxylin–eosin staining and the decrease of serum transaminase levels confirmed that salmon sperm DNA could significantly improve hepatocyte injury in a dose‐dependent manner.

When acute liver injury occurs, a large number of hepatocytes play a role in apoptosis and necrosis, if the excessive death of cells exceeds the clearance capacity, that is, apoptosis is enhanced, it will further cause cell necrosis. Bax and Bcl‐2 are a pair of apoptosis‐related proteins that antagonize each other, Bax promotes apoptosis and Bcl‐2 inhibits apoptosis (Hoshyar et al., 2013), Caspase 3 is a key protease in the process of apoptosis and a key effector downstream of many apoptotic pathways. The results showed that compared with the Normal group, the expression of Bax and Caspase 3 in the Model group was upregulated, while the expression of Bcl‐2 was downregulated, and the percentage of TUNEL positive cells was significantly increased. At the same time, compared with the Model group, salmon sperm DNA group could reduce the percentage of TUNEL positive cells in a dose‐dependent manner. In short, salmon sperm DNA has an inhibitory effect on CCl_4_‐induced apoptosis in mice.

CCl_4_ metabolism produces a large number of free radicals, leading to lipid peroxidation. Oxidative stress induced by free radicals is one of the important mechanisms of CCl_4_‐induced liver injury (Torok, 2016). Nrf2/ARE pathway is the most important antioxidant stress pathway in the body. This study confirmed that salmon sperm DNA activates the Nrf2/ARE signaling pathway. It decreases the expression of keap1 protein, increases the protein expression of Nrf2 and NQO1, and improves the state of oxidative stress; furthermore, the levels of antioxidants SOD, GSH‐Px, and GSH increased while the content of MDA decreased.

Inflammatory reaction is one of the important pathological mechanisms of CCl_4_‐induced liver injury (Chen et al., 2016). Oxidative stress induced by CCl_4_ can not only indirectly or directly damage hepatocytes, but also cause the release of damage molecular‐related patterns, lead to the activation of inflammatory cells, and produce pro‐inflammatory cytokines such as TNF‐α and IL‐6 to aggravate liver injury (Kiso et al., 2012). The results showed that salmon sperm DNA could significantly reduce the levels of TNF‐α and IL‐6 in a dose‐dependent manner.

## 5 CONCLUSION

In conclusion, our experimental results showed that the obviously protective effects of salmon sperm DNA against CCl_4_‐induced acute liver injury have contributed to the inhibition of oxidative stress, attenuation of hepatocyte apoptosis, and reduction of the inflammatory response, probably through Nrf2/ARE and mitochondrial apoptosis pathway on acute CCl_4_‐induced liver injury in mice, and also have anti‐inflammatory effects. According to the conclusion of the hepatoprotective effect of salmon sperm DNA obtained from the results of animal experiments, more detailed studies on the mechanism will be carried out at the cellular and molecular level in the future, which will provide a scientific basis for the utilization and development of waste nucleic acid resources in aquatic products.

## AUTHOR CONTRIBUTIONS

*Xinyi Huang*: Conceptualization, Methodology, Software, Formal analysis, Data curation, Writing – original. *Xu Chu*: Conceptualization, Methodology, Data curation. *Yingying Tian*: Data curation, Writing – review & editing. *Yuhan Xue*: Formal analysis, Writing – review & editing. *Lei Zhang*: Methodology, Writing – review & editing. *Jing Li*: Supervision Writing – review & editing. *Hu Hou*: Supervision, Writing – review & editing. *Ping Dong*: Conceptualization, Writing – review & editing, Funding acquisition, Supervision. *Jingfeng Wang*: Conceptualization, Methodology, Writing – review & editing.

## CONFLICT OF INTEREST

The authors declare that they have no known competing financial interests or personal relationships that could have appeared to influence the work reported in this paper.

## ETHICAL APPROVAL

The Ethics Committee approved this study of Experimental Animal Care at Ocean University of China (Certificate No. SPXY2020040110). Three‐week‐old male ICR mice were purchased from Jinan Pengyue Experimental Animal Breeding Co. Ltd. (Jinan, China; Licensed ID: 1107261911000276).
